# Blockade of novel immune checkpoints and new therapeutic combinations to boost antitumor immunity

**DOI:** 10.1186/s13046-022-02264-x

**Published:** 2022-02-14

**Authors:** Adrià Archilla-Ortega, Carla Domuro, Juan Martin-Liberal, Purificación Muñoz

**Affiliations:** 1 Aging and Cancer Group, Oncobell Program, Bellvitge Biomedical Research Institute (IDIBELL), Av. Gran Vía de L’Hospitalet 199-203, 08907 Barcelona, Spain; 2 Medical Oncology Department, Catalan Institute of Oncology (ICO) Hospitalet, IDIBELL, Barcelona, Spain

**Keywords:** Immunotherapy, Immune checkpoint, Cytotoxic T lymphocytes, NK cells, Tumor microenvironment

## Abstract

Immunotherapy has emerged as a promising strategy for boosting antitumoral immunity. Blockade of immune checkpoints (ICs), which regulate the activity of cytotoxic T lymphocytes (CTLs) and natural killer (NK) cells has proven clinical benefits. Antibodies targeting CTLA-4, PD-1, and PD-L1 are IC-blockade drugs approved for the treatment of various solid and hematological malignancies. However, a large subset of patients does not respond to current anti-IC immunotherapy. An integrative understanding of tumor-immune infiltrate, and IC expression and function in immune cell populations is fundamental to the design of effective therapies. The simultaneous blockade of newly identified ICs, as well as of previously described ICs, could improve antitumor response. We review the potential for novel combinatory blockade strategies as antitumoral therapy, and their effects on immune cells expressing the targeted ICs. Preclinical evidence and clinical trials involving the blockade of the various ICs are reported. We finally discuss the rationale of IC co-blockade strategy with respect to its downstream signaling in order to improve effective antitumoral immunity and prevent an increased risk of immune-related adverse events (irAEs).

**Supplementary Information:**

The online version contains supplementary material available at 10.1186/s13046-022-02264-x.

## Background

Tumor growth involves a complex interplay between tumor, immune, and stromal cells, and extracellular matrix components. In the last decade, the relevance of the tumor-immune microenvironment and its direct impact on patients’ clinical outcome has become widely acknowledged [1]. The host immune system is primed to identify and kill malignantly transformed cells to prevent tumorigenesis and tumor growth. Cytotoxic T lymphocytes (CTLs) and natural killer (NK) cells are immune cell populations responsible for immunosurveillance and, when required, for eliminating target cells. Tumor cells can be identified by CTLs as altered cells by the expression of neoantigens displayed by the major histocompatibility complex (MHC) [2]. Tumor cells expressing low levels of MHC molecules can become invisible to T cells and may escape T-cell immune control. In these cases, NK cells can identify and target cancer cells that lack MHC expression. However, tumor immune evasion, defined as the ability of tumor cells to evade the host’s immune response, happens during tumorigenesis and tumor growth. Multiple activating and inhibiting mechanisms tightly regulate the effector function of CTLs and NK cells to prevent autoimmune events and preserve tissue homeostasis. In this regard, immune checkpoints (ICs) are signaling pathways that modulate the immune response. CTLs and NK cells can express IC receptors that, when interacting with IC ligands, activate IC signaling pathways, blocking their cytotoxic activity [3]. These IC ligands can be expressed by immunosuppressive cells, including M2-like macrophages, myeloid-derived suppressor cells (MDSCs), and T-regulatory (Treg) cells, as well as cancer cells. The continuous interaction between IC ligands and their respective IC receptors expressed by CTLs and NK, help produce a dysfunctional state in these immune cells known as exhaustion. Tumors avoid antitumoral immunity by upregulating the expression of IC ligands and recruiting immunosuppressive cells, which give rise to an immunosuppressive tumor microenvironment (TME). Tumors with a strong immunosuppressive TME have been associated with impaired immune cytotoxicity, are more aggressive, and have a poor prognosis [4].

Immunotherapy is based on stimulating the host immune system to elicit a response against cancer cells. Pharmacological blockade of the interaction between ICs and their ligands with IC inhibitors has been identified as a promising strategy for restoring immune cytotoxic activity [5]. In recent years, T cell-mediated cytolysis has been the focus of major efforts to modulate antitumor cytotoxicity as therapy. However, NK cells are another immunotherapy target for boosting antitumor response. Importantly, the IC receptors expressed by NK and T cells can be expressed by other immune cell populations, and their blockade may also modulate the function of those populations. Here, we describe the various IC receptors expressed by T, NK, and other immune cells, and their biological function. We also analyze the antitumoral activity of IC blockade therapies, as single agents or in combination, for cancer treatment.

## Main text

### Effector cells: cytotoxic T lymphocytes and natural killer cells

CTLs and NK cells are the two major immune populations that are able to eliminate malignant cells. CTLs participate in the adaptive immune response while NK cells are part of the innate immune system. Cytotoxicity arises by two pathways: Perforin/Granzyme B/Granulysin-related lysis, and death receptor-induced apoptosis. Although CTLs and NK cells act in a mechanistically similar fashion, the regulation of the activity of these immune cells, and the recognition of the targets differ. CTL cytotoxicity is acquired after T cell activation upon antigen presentation by antigen presenting cells (APCs) —mainly dendritic cells (DCs) — whereas NK cells lyse target cells without antigen presentation [6]. When activated, CTLs and NK cells both secrete interferon (IFN)-γ and tumor necrosis factor (TNF)-α, which stimulate a pro-inflammatory immune response. Antitumoral effects have been extensively ascribed to these two immune cell populations, highlighting the relevance of comprehensively understanding the activation and inhibition mechanisms that regulate their cytotoxic activity against cancer cells by pharmacological strategies.

CTLs are defined as activated effector CD3^+^ CD8^+^ T lymphocytes and recognize target cells via the interaction between polyclonally rearranged T-cell receptor (TCR) with a peptide/MHC class I complex. Naïve CD8^+^ T cells interact with APCs and, upon the correct presentation of the peptide-MHC class I complex, TCR signaling causes the formation of a stabilization complex between T cells and the APC. To become fully activated, the co-stimulatory receptor CD28 must interact with its ligands, CD80 (B7.1) and CD86 (B7.2). The activity of T cells is determined by the balance of positive and negative signals from co-activator and co-inhibitory receptors when they recognize their target. To eliminate target cells, CTLs produce a stabilization complex, after which, lytic granules are secreted. Perforin forms pores in the extracellular membrane of the target cells, allowing Granzyme B and Granulysin to enter the cytosol and induce apoptosis, while membrane-bound FasL binds to its receptor Fas, inducing apoptosis in an independent manner [6].

Human NK cells are phenotypically defined as CD3^−^ CD56^+^ lymphocytes. Two functionally distinct subsets of NK cells have been defined on the basis of their levels of expression of CD56 and CD16 (also known as Fcγ receptor III). NK cells with high-density expression of CD56 (CD56^bright^) and CD16^−^ are mostly found in lymph nodes and have a great ability to release immune-modulating cytokines such as IFN-γ and TNF-α. On the other hand, low-density expression of CD56 (CD56^dim^) CD16^+^ NK cells mostly occurs in peripheral blood, where it presents a more cytotoxic phenotype characterized by high levels of Perforin and Granzyme B expression [7]. Cytotoxic NK cell activity is independent of foreign antigens presented by MHC molecules. The balance between activation and inhibition signals, which NK cells sense through multiple innate receptors, allows the cells to respond to alterations such as cellular stress, cellular transformation, and malignancy. When activated, NK cells form a stabilization complex similarly to CTLs and release cytotoxic granules.

**Fig. 1:**
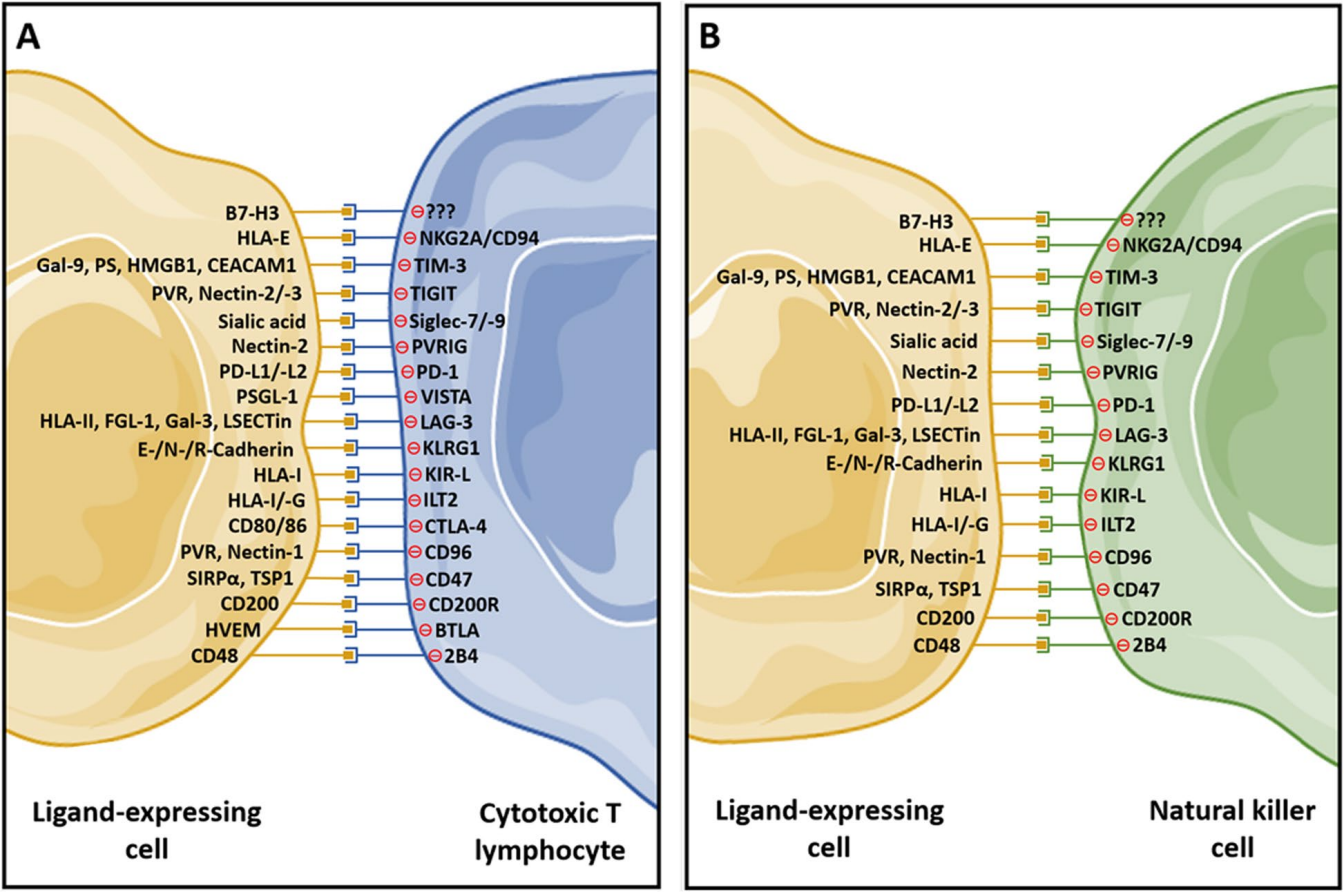
Coinhibitory receptors expressed by cytotoxic T lymphocytes (**A**) and natural killer cells (**B**) and their ligands. Cytotoxic T lymphocytes and natural killer cells can express multiple coinhibitory receptors, known as immune checkpoints, that produce downstream inhibitory signals when activated upon binding to their ligands. Note that not all ICs are expressed simultaneously by cytotoxic T lymphocytes or NK cells

CTL and NK-cell activity is tightly controlled to preserve antigenic self-tolerance. Autoreactive T-cell clones are eliminated in the thymus by a process known as central tolerance. Also, a peripheral regulation of the cytotoxic response is essential to avoid inappropriate responses to self-antigens. The release of immunosuppressive molecules by M2-like macrophages and Treg cells plays a key role in establishing immune self-tolerance [8]. Activated CTLs and NK cells upregulate the expression of multiple coinhibitory receptors, known as ICs receptors, which downregulate their cytotoxic activity when binding to their ligands, ensuring the precise regulation of their effector function (Fig. 1). Although self-tolerance mechanisms are tightly regulated, T-cell exhaustion occurs and is often observed in tumors and chronic infections [9]. NK cells can present a similar exhausted phenotype that is characterized by stronger expression of coinhibitory receptors and weaker expression of activating receptors [10]. Tumor-infiltrating lymphocytes (TILs) and tumor-infiltrating NK cells exhibit enhanced expression of IC receptors [5, 10]. This has boosted interest in understanding how these coinhibitory receptors function in order to therapeutically block them. The best characterized IC receptors are the cytotoxic T-lymphocyte-associated molecule 4 (CTLA-4) and the programmed cell death protein 1 (PD-1), but many other ICs play key central roles in controlling CTL and NK cell effector functions (Table 1).

**Table 1:** Expression of coinhibitory receptors and their ligands in immune cell populations

Coinhibitory receptor	Expression in human CTLs	Expression in human NK cells	Expression in other immune cell populations	Ligand-expressing cells
**2B4 (CD244, SLAMF4)**	Subsets of CTLs and increased in tumor-infiltrating CTLs	Subsets of NK cells	DCs, monocytes, MDSCs and basophils	CD48 (SLAMF2): Lymphocytes, DCs, monocytes, macrophages, endothelial cells and tumor cells
**BTLA**	Subsets of CTLs and increased in tumor-infiltrating CTLs	Not expressed	B cells and DCs	HVEM: T cells, B cells, NK cells, DCs, monocytes and tumor cells
**CD200R**	Subsets of CTLs	Subsets of NK cells	Th cells, B cells, DCs, macrophages and neutrophils	CD200: T cells, B cells, DCs, endothelial cells and tumor cells
**CD47 (IAP)**	Subsets of CTLs	Subsets of NK cells	Expressed ubiquitously in human cells	SIRPα: DCs, macrophages, monocytes and granulocytes TSP1: Endothelial cells, monocytes, macrophages, granulocytes and cancer cells
**CD96 (TACTILE)**	Subsets of CTLs and increased in tumor-infiltrating CTLs	Subsets of NK cells and increased in tumor-infiltrating NK cells	Treg cells	PVR (CD155. Necl-5): DCs, neutrophils, macrophages and tumor cells Nectin-1 (PVRL1, CD111): DCs and tumor cells
**CTLA-4**	Subsets of CTLs and increased in tumor-infiltrating CTLs	Unclear	Th cells and Treg cells	CD80 (B7–1): B cells, T cells, DCs, macrophages and tumor cells CD86 (B7–2): B cells, T cells, DCs, macrophages and tumor cells
**ILT2 (LIR1)**	Subsets of CTLs and increased in tumor-infiltrating CTLs	Subsets of NK cells and increased in tumor-infiltrating NK cells	Th cells, B cells, DCs and MDSCs	HLA-G: Tumor cells HLA-I (A/B/C): Ubiquitously in human cells
**KIR-L**	Subsets of CTLs and increased in tumor-infiltrating CTLs	Subsets of NK cells and increased in tumor-infiltrating NK cells	Th cells	HLA-I (A/B/C): Ubiquitously in human cells
**KLRG1**	Subsets of CTLs and increased in tumor-infiltrating CTLs	Subsets of NK cells	Not expressed	E-cadherin: Epithelial cells and cancer cells N-cadherin: Mesenchymal cells and cancer cells R-cadherin: Neural tissue
**LAG-3**	Subsets of CTLs and increased in tumor-infiltrating CTLs	Subsets of NK cells	Th cells, Treg cells, B cells and DCs	HLA-II: DCs, macrophages, B cells, neutrophils, fibroblasts and tumor cells FGL-1: Tumor cells Gal-3: Fibroblasts LSECtin: Tumor cells
**NKG2A/CD94**	Subsets of CTLs and increased in tumor-infiltrating CTLs	Subsets of NK cells and increased in tumor-infiltrating NK cells	Not expressed	HLA-E: Ubiquitously in human cells and increased in tumor cells
**PD-1**	Subsets of CTLs and increased in tumor-infiltrating CTLs	Expressed in tumor-infiltrating NK cells	Th cells, Treg cells, B cells, macrophages and DCs	PD-L1 (B7-H1): Treg cells, B cells, DCs, macrophages, monocytes, MDSCs and tumor cells. PD-L2 (B7-DC): DCs, macrophages, B cells and tumor cells.
**PVRIG (CD112R)**	Subsets of CTLs and induced in tumor-infiltrating CTLs	Subsets of NK cells and increased in tumor-infiltrating NK cells	Not expressed	Nectin-2 (PVRL2, CD112): DCs, endothelial cells, and tumor cells
**Siglec-7/−9**	Siglec-7: Subsets of CTLs, Siglec-9: Subsets of CTLs and induced in tumor-infiltrating CTLs	Siglec-7: Subsets of NK cells, Siglec-9: Subsets of NK cells	Siglec-7: Macrophages, monocytes and mast cells Siglec-9: DCs, macrophages, monocytes and neutrophils	Sialic acid-containing ligands: Glycoproteins expressed by human cells. Hypersialylation, xenosialylation, and sialic acid alterations in tumor cells
**TIGIT**	Subsets of CTLs and increased in tumor-infiltrating CTLs	Subsets of NK cells and increased in tumor-infiltrating NK cells	Th cells and Treg cells	PVR (CD155, Necl-5): DCs, neutrophils, macrophages and tumor cells Nectin-2 (PVRL2, CD112): DCs, endothelial cells, and tumor cells Nectin-3 (PVRL3, CD113): DCs, endothelial cells, T cells and tumor cells
**TIM-3**	Subsets of CTLs and increased in tumor-infiltrating CTLs	Subsets of NK cells and increased in tumor-infiltrating NK cells	Th cells, Treg cells, DCs, macrophages and monocytes	Galectin-9: T cells, Treg cells, NK cells, B cells, macrophages, endothelial cells and mast cells and tumor cells. PS: Inner-cell membrane phospholipid of human cells. HMGB1: NK cells, DCs, macrophages and tumor cells CEACAM1: T cells, B cells, NK cells, neutrophils, monocytes and tumor cells
**Undiscovered B7-H3 receptor**	/	/	/	B7-H3: T cells, B cells, NK cells, monocytes and tumor cells
**VISTA (PD-1H)**	Subsets of CTLs and increased in tumor-infiltrating CTLs	Not expressed	Th cells, Treg cells, DCs, macrophages, monocytes, neutrophils and basophils	PSGL-1: T cells, B cells, DCs, macrophages, monocytes, endothelial cells, and neutrophils

*CTLs* cytotoxic T lymphocytes, *NK* natural killer, *DCs* dendritic cells, *MDSCs* myeloid-derived suppressor cells, *BTLA* B and T cell lymphocyte attenuator, *HVEM* herpesvirus entry mediator, *Th* T-helper, *SIRPα* signal regulatory protein alpha, *TSP1* thrombospondin-1, *Treg* T-regulatory, *ILT2* immunoglobulin-like transcript-2, *KIR-L* killer immunoglobulin-like receptor long cytoplasmic tail, *LAG3* lymphocyte activation gene-3, *FGL-1* fibrinogen-like protein 1, *Gal-3* galectin-3, *NKG2A* natural killer group 2A, *PD-1* programmed cell death protein 1, *PD-L1* programmed death-ligand 1, *PD-L2* programmed cell death 1 ligand 2, *TIGIT* T cell immunoglobulin and ITIM domain, *TIM-3* T-cell immunoglobulin and mucin-domain containing-3, *PS* phosphatidylserine (PS), *HMGB1* high-mobility group protein 1, *CEACAM1* carcinoembryonic antigen-related cell adhesion molecule 1, *VISTA* V-domain Ig suppressor of T cell activation, *PSGL-1* P-selectin glycoprotein ligand 1

### Approved IC inhibitors for cancer treatment

IC signaling inhibition can be exploited as a therapeutic strategy for treating cancer [11, 12]. CTLA-4 and PD-1 were the first ICs to be blocked in preclinical models addressing cancer therapy. Several IC blockade antibodies were approved by the US Food and Drug Administration (FDA) and the European Medicines Agency (EMA), and are currently used to treat a variety of tumor types (Table 2). However, we are far from fully exploiting the potential of this therapeutic strategy. CTLs and NK cells may be modulated by anti-IC drugs by direct mechanisms when the effector cells express the targeted IC, and/or by indirect mechanisms, when blockade of the IC alters the immunomodulatory functions of other immune cell populations. While the currently approved IC blockade drugs produce a good clinical response in some patients, a percentage of patients show short-duration response or even no response at all (Table 3). Furthermore, late relapses occur in subsets of patients who initially responded to IC blockade therapy [13], which has sparked interest in targeting other coinhibitory receptors to find new therapeutic combinations to treat non-responsive tumors.

**Table 2:** Immune checkpoint inhibitors approved for clinical use up to 2021, according to www.fda.gov

Target	Drug name	Indication	Brand name (company)
**CTLA-4**	Ipilimumab	Melanoma; metastatic melanoma	Yervoy (Bristol-Myers Squibb Co.)
**CTLA-4 + PD-1**	Ipilimumab + nivolumab	HCC; metastatic CRC; metastatic melanoma; metastatic RCC; metastatic NSCLC; malignant pleural mesothelioma	Yervoy + Opdivo (Bristol-Myers Squibb Co.)
**PD-1**	Nivolumab	HCC; Hodgkin lymphoma; metastatic CRC; metastatic melanoma; melanoma; metastatic RCC; metastatic urothelial carcinoma; metastatic HNSCC; metastatic SCLC; metastatic NSCLC; ESCC	Opdivo (Bristol-Myers Squibb Co.)
**PD-1**	Pembrolizumab	HCC; Merkel cell carcinoma; NSCLC; metastatic squamous NSCLC; RCC; melanoma; metastatic gastric cancer; metastatic gastroesophageal junction adenocarcinoma; metastatic urothelial carcinoma; metastatic cervical cancer; Hodgkin lymphoma; metastatic NSCLC; metastatic endometrial cancer; CRC; NMIBC; pancreatic cancer; primary mediastinal B-cell lymphoma; metastatic HNSCC; metastatic tumor mutational burden-high solid tumors; metastatic cSCC, metastatic TNBC	Keytruda (Merck & Co. Inc.)
**PD-1**	Cemiplimab	Metastatic cSCC	Libtayo (Regeneron Pharmaceuticals Inc. / Sanofi-Aventis SA)
**PD-L1**	Atezolizumab	Metastatic TNBC; metastatic NSCLC; SCLC; metastatic urothelial cancer; HCC; metastatic melanoma	Tecentriq (Genentech Inc. / Roche Registration Ltd.)
**PD-L1**	Durvalumab	SCLC; metastatic urothelial cancer; NSCLC	Imfinzi (AstraZeneca Pharmaceuticals LP)
**PD-L1**	Avelumab	Metastatic RCC; metastatic urothelial cancer; Merkel cell carcinoma	Bavencio (Merck KGaA / Pfizer Inc.)

*HCC* hepatocellular carcinoma, *CRC* colorectal cancer, *RCC* renal cell carcinoma, *NSCLC* non-small-cell lung cancer, *HNSCC* head and neck squamous cell carcinoma, *SCLC* small-cell lung cancer, *ESCC* esophageal squamous cell carcinoma, *NMIBC* non-muscle invasive bladder cancer, *cSCC* cutaneous squamous cell carcinoma, *TNBC* triple-negative breast cancer

**Table 3:** Summary of the differences in the application of immune checkpoint inhibitors against common tumors, according to FDA approval information

Condition	Drug	ORR (%)	mPFS (months)	mOS (months)	Grade 3–5 AE (%)	Approval time	Key trials
**Melanoma**	Ipilimumab	16.8	2.70	11.2	11	03/2011	NCT00324155
	Ipilimumab + nivolumab	60	8.50	Not reached	62	10/2015	CheckMate-067
	Pembrolizumab	37	5.00	> 24	< 10	09/2014	KeyNote-001
	Nivolumab	32	4.70	17.3	9	12/2014	CheckMate-037
	Atezolizumab	60	15.10	16.1	33.5	07/2020	IMspire150
**NSCLC**	Nivolumab	20	3.50	9.2	7	03/2015	CheckMate-063; CheckMate-017
	Ipilimumab + nivolumab	36	5.1	17.1	58	05/2020	CheckMate-227
	Pembrolizumab	19.4	4.00	12.7	< 10	10/2015	KeyNote-001; KeyNote-010
	Atezolizumab	17	2.70	12.6	11	10/2016	Poplar (NCT01903993)
	Durvalumab	28.4	16.80	23.2	29.9	02/2018	NCT02125461
	Cemiplimab	39	8.20	Not reached	28	02/2021	NCT03088540
**RCC**	Nivolumab	25	4.60	25	19	11/2015	CheckMate-025
	Ipilimumab + nivolumab	41.6	11.6	Not reached	65	04/2018	CheckMate-214
	Pembrolizumab	59.3	15.10	Not reached	75.8	04/2019	KeyNote-426
	Avelumab	51.4	13.80	11.6	71.2	05/2019	JAVELIN Renal 101
**HCC**	Nivolumab	14.3	4.00	15	25	09/2017	CheckMate-040
	Ipilimumab + nivolumab	33	8.3	17.5	37	03/2020	Checkmate-040
	Pembrolizumab	17	4.9	12.9	26	11/2018	KeyNote-224
	Atezolizumab	65	6.80	Not evaluated	56.5	05/2020	IMbrave150
**SCLC**	Nivolumab	12	1.4	5.6	45	08/2018	CheckMate-032
	Atezolizumab	60.2	5.20	12.3	37	03/2019	Impower133
	Pembrolizumab	19	2.0	8.7	31	06/2019	KeyNote-158; KeyNote-028
	Durvalumab	68	5.10	13	62	03/2020	CASPIAN (NCT03043872)
**CRC**	Nivolumab	32	14.30	5.6	45	08/2017	CheckMate-142
	Ipilimumab + nivolumab	55	12	Not evaluated	20	07/2018	CheckMate-8HW
	Pembrolizumab	43.8	16.50	13.7	22	06/2020	KeyNote-177

*ORR* overall rate response, *mPFS* median progression free survival, *mOS* median overall survival, *AE* adverse events, *NSCLC* non-small-cell lung cancer, *RCC* renal cell carcinoma, *HCC* hepatocellular carcinoma, *SCLC* small-cell lung cancer, *CRC* colorectal cancer

#### CTLA-4 blockade

CTLA-4, which binds CD80 and CD86 ligands, was the first IC to be described in T cells [11]. CD80 and CD86 are also ligands of the T cell co-activator receptor CD28. CTLA-4 has a high sequence similarity to that of CD28 and competes, with higher affinity, to bind CD80 and CD86. CTLA-4 regulates early stages of CD8^+^ T cell activation, ensuring tolerance of self-antigens in the lymph nodes. Mechanistically, CTLA-4 indirectly limits CD28 signaling by capturing and sequestering CD80 and CD86 from ligand-expressing cells through a process named trans-endocytosis [14]. Genetic ablation of CTLA-4 increases early lethality in mice due to lymphoproliferation [5]. Blockade experiments demonstrated that in vitro and in vivo anti-CTLA-4 treatment enhance T-cell proliferation [15]. CTLA-4 is also expressed by Treg cells, and its signaling enhances their suppressive functions [16]. In addition, CD4^+^ T cells expressing CTLA-4 exhibit lower rates of proliferation [17]. The role of CTLA-4 in NK cells remains to be elucidated. While some studies indicated that CTLA-4 is expressed in mouse and human NK cells, and that it regulates their effector functions, others reported that human NK cells are not affected by the CD28/CTLA-4 axis [18].

In vivo administration of anti-CTLA-4 antibody increases antitumor immunity, causing pre-established tumors to be rejected [11]. Further experiments demonstrated that blockade of CTLA-4 stimulates the CD8^+^ T cell cytotoxic response against tumor cells [5]. Importantly, the complete antitumoral response provoked by CTLA-4 blockade requires Treg cells. CTLA-4 conditional knockout (KO) in Treg cells reduces tumor growth [15]. Blockade of CTLA-4 accelerates Th1 proliferation and increases interleukin (IL)-2 production. While no direct function of anti-CTLA-4 in NK cells has yet been clearly identified, in vivo blockade of CTLA-4 might enhance NK cell activity indirectly by inhibiting Treg immunosuppressive activity and enhancing Th1 pro-inflammatory function [18]. Finally, the concomitant blockade of CTLA-4 and PD-1 signaling enhances antitumor response and increases the cytotoxic activity of CD8^+^ T cells in preclinical models [19].

CTLA-4 was the first IC receptor to be clinically targeted. In 2011, ipilimumab, an anti-CTLA-4 monoclonal antibody (mAb), was approved by the FDA and the EMA for treating patients with advanced melanoma. At present, ipilimumab is the only anti-CTLA-4 mAb approved for clinical use. It has value as a monotherapy for melanoma in adjuvant and metastatic settings, or combined with nivolumab, an anti-PD-1 antibody, for patients suffering several advanced cancer types (Table 2). Several clinical trials are testing the efficacy of anti-CTLA-4 combined with other anti-ICs in a variety of tumor types (Table 4).

**Table 4:** Clinical-stage development of monotherapies and combinatory therapies with immune checkpoint inhibitors in 2021, according to www.clinicaltrials.gov

Target	Drug name	Indication	Status
**CTLA-4 + PD-1**	Ipilimumab + nivolumab	NSCLC (NCT03351361) (NCT02864251) (NCT02477826) (NCT02869789) (NCT03391869) (NCT04026412) (NCT02998528) (NCT03215706); gastric cancer; GEJ cancer (NCT02872116) (NCT03604991); HNSCC (NCT03700905); metastatic HNSCC (NCT02741570); melanoma (NCT02905266) (NCT02599402) (NCT03068455) (NCT02388906); SCLC (NCT02538666); RCC (NCT03793166) (NCT03873402) (NCT03937219) (NCT03138512) (NCT04513522); esophageal cancer (NCT03143153); sarcoma (NCT04741438); glioblastoma (NCT02017717) (NCT04396860); squamous cell lung cancer (NCT02785952); metastatic urothelial cancer (NCT03036098); metastatic prostate cancer (NCT03879122)	Phase III
**CTLA-4 + PD-1**	Ipilimumab + pembrolizumab	Metastatic NSCLC (NCT03302234); metastatic melanoma (NCT01866319); solid tumors (NCT03755739)	Phase III
**CTLA-4 + PD-1**	Ipilimumab + REGN2810	NSCLC (NCT03515629) (NCT03409614)	Phase III
**CTLA-4 + PD-L1**	Tremelimumab + durvalumab	Advanced solid tumors (NCT03084471); HNSCC (NCT02369874); NSCLC (NCT02453282); SCLC (NCT03703297); metastatic NSCLC (NCT02542293) (NCT02352948) (NCT03164616); metastatic urothelial cancer (NCT03682068); urothelial cancer (NCT02516241); metastatic HNSCC (NCT02551159); HCC (NCT03298451); squamous cell lung cancer (NCT02154490); RCC (NCT03288532); SCLC (NCT03043872)	Phase III
**PD-1 + LAG-3**	Nivolumab + relatlimab	Metastatic melanoma (NCT03470922)	Phase III
**PD-1 + LAG-3 + B7-H3**	MGD013 + enoblituzumab	Gastric cancer; GEJ cancer (NCT04082364), metastatic HNSCC (NCT04129320)	Phase III
**PD-1 + TIGIT**	Tislelizumab + BGB-A1217	NSCLC (NCT04746924)	Phase III
**PD-1 + TIGIT**	Zimberelimab + AB154	Metastatic NSCLC (NCT04736173)	Phase III
**PD-1 + B7-H3**	MGA012 + enoblituzumab	Metastatic HNSCC (NCT04129320)	Phase III
**PD-L1 + TIGIT**	Atezolizumab + tiragolumab	NSCLC (NCT04294810) (NCT04513925); SCLC (NCT04256421) (NCT04665856); esophageal squamous cell carcinoma (NCT04540211) (NCT04543617);	Phase III
**LAG-3 + PD-1**	Relatlimab + nivolumab	Multiple solid tumors (NCT01968109); CRC (NCT03642067); metastatic soft-tissue sarcoma (NCT04095208); HNSCC (NCT04080804); NSCLC (NCT04205552) (NCT02750514); RCC (NCT02996110); gastric cancer (NCT02935634); metastatic melanoma (NCT04552223) (NCT03743766) (NCT03724968); solid tumors (NCT03607890); HCC (NCT04567615); metastatic basal cell carcinoma (NCT03521830); HNSCC (NCT04326257); metastatic NSCLC (NCT04623775); metastatic CRC (NCT03867799); GEJ adenocarcinoma (NCT03704077) (NCT03662659) (NCT03610711) (NCT04062656); advanced cancers (NCT03459222) (NCT02488759); metastatic ovarian cancer (NCT046111269); melanoma (NCT02519322); multiple myeloma (NCT04150965)	Phase II
**LAG-3 + PD-1**	BI-754111 + BI-754091	Metastatic solid tumors (NCT03697304)	Phase II
**LAG-3 + PD-1**	REGN3767 + cemiplimab	Breast cancer (NCT01042379); metastatic solid tumors (NCT04706715)	Phase II
**LAG-3 + PD-1**	LAG525 + spartalizumab	TNBC (NCT03499899); advanced malignancies (NCT03365791) (NCT02460224); melanoma (NCT03484923)	Phase II
**LAG-3 + PD-1 + TIM-3**	INCAGN02385 + INCMGA00012 + INCAGN02390	Advanced malignancies (NCT04370704)	Phase II
**LAG-3 + PD-1**	MK-4280 + pembrolizumab	Hodgkin lymphoma; non-Hodgkin lymphoma (NCT03598608); advanced NSCLC (NCT03516981)	Phase II
**TIM-3**	MBG453	Myelodysplastic syndromes; chronic myelomonocytic leukemia (NCT04266301)	Phase III
**TIM-3**	MBG453	AML (NCT04150029) (NCT04623216); advanced solid tumors (NCT02608268); myelofibrosis (NCT04097821)	Phase II
**TIM-3 + PD-1**	BMS-986258 + nivolumab	Solid tumors (NCT03446040)	Phase II
**TIM-3 + PD-1**	BGB-A425 + tislelizumab	Solid tumors (NCT03744468)	Phase II
**TIM-3 + PD-1**	TSR-022 + TSR-042	Liver cancer (NCT03680508); melanoma (NCT04139902)	Phase II
**TIGIT + PD-L1**	Tiragolumab + atezolizumab	Cervical cancer (NCT04300647); gastric adenocarcinoma; GEJ adenocarcinoma; esophageal carcinoma (NCT03281369); urothelial carcinoma (NCT03869190); pancreatic adenocarcinoma (NCT03193190); NSCLC (NCT03563716); metastatic NSCLC (NCT04619797); metastatic HNSCC (NCT04665843); SCLC (NCT04308785); HNSCC (NCT03708224); liver cancer (NCT04524871)	Phase II
**TIGIT + PD-1**	AB154 + zimberelimab	NSCLC (NCT04262856)	Phase II
**TIGIT**	BMS-986207	Multiple myeloma (NCT04150965)	Phase II
**TIGIT + PD-1**	BMS-986207 + nivolumab	Solid tumors (NCT02913313) (NCT04570839)	Phase II
**PVRIG + PD-1**	COM701 + nivolumab	Advanced solid tumors (NCT03667716) (NCT04570839)	Phase I
**KIR2DL1 + KIR2DL2 + KIR2DL3**	Lirilumab	AML (NCT01687387); chronic lymphocytic leukemia (NCT02481297); refractory AML (NCT02399917)	Phase II
**KIR2DL1 + KIR2DL2 + KIR2DL3 + PD-1**	Lirilumab + nivolumab	HNSCC (NCT03341936); metastatic malignancies (NCT03347123); multiple myeloma (NCT01592370); refractory tumors (NCT02813135)	Phase II
**KIR2DL1 + KIR2DL2 + KIR2DL3 + PD-1 + CTLA-4**	Lirilumab + nivolumab + ipilimumab	Advanced solid tumors (NCT01714739)	Phase II
**KIR3DL2**	IPH4102	Advanced T cell lymphoma (NCT03902184).	Phase II
**NKG2A**	Monalizumab	Metastatic HNSCC (NCT04590963)	Phase III
**NKG2A**	Monalizumab	Metastatic HNSCC (NCT02643550) (NCT03088059); breast cancer (NCT04307329); chronic lymphoid leukemia (NCT02557516)	Phase II
**NKG2A + PD-L1**	Monalizumab + durvalumab	CRC (NCT04145193); solid tumors (NCT02671435); NSCLC (NCT03822351) (NCT038223519) (NCT03833440)	Phase II
**CD200**	Samalizumab	AML (NCT03013998), multiple myeloma (NCT00648739)	Phase II
**CD47**	Magrolimab	Myelodysplastic syndrome (NCT04313881); AML (NCT04778397)	Phase III
**CD47 + PD-L1**	Magrolimab + atezolizumab	Metastatic urothelial carcinoma (NCT03869190)	Phase II
**CD47**	Magrolimab	Solid tumors (NCT02953782); refractory B-cell non-Hodgkin lymphoma (NCT02953509); myeloid malignancies (NCT04778410)	Phase II
**CD47**	RRX-001	SCLC (NCT03699956)	Phase III
**CD47**	RRX-001	Solid tumors (NCT02489903); metastatic CRC (NCT02096354)	Phase II
**BTLA**	JS004	Advanced solid tumors (NCT04278859)	Phase I
**BTLA**	TAB004	Advanced solid malignancies (NCT04137900)	Phase I
**VISTA**	JNJ-61610588	Advanced solid tumors (NCT02671955)	Phase I
**VISTA + PD-L1**	CA170	Advanced solid tumors and lymphomas (NCT02812875)	Phase I
**B7-H3**	Enoblituzumab	Metastatic HNSCC (NCT04129320)	Phase III
**B7-H3**	Enoblituzumab	Prostate cancer (NCT02923180)	Phase II
**B7-H3**	131I-omburtamab	Neuroblastoma; central nervous system metastases; leptomeningeal metastases (NCT03275402)	Phase III
**B7-H3**	177Lu-DTPA-omburtamab	Medulloblastoma (NCT04167618); solid tumors (NCT04315246)	Phase II
**B7-H3**	DS-7300a	Advanced solid tumors (NCT04145622)	Phase II
**B7-H3 + PD-1**	MGC018 + MGC012	Advanced solid tumors (NCT03729596)	Phase II

*NSCLC* non-small-cell lung cancer, *GEJ* gastroesophageal junction, *HNSCC* head and neck squamous cell carcinoma, *SCLC* small-cell lung cancer, *RCC* renal cell carcinoma, *HCC* hepatocellular carcinoma, *MIBC* muscle invasive bladder cancer, *ESCC* esophageal squamous cell carcinoma, *TNBC* triple-negative breast cancer, *CRC* colorectal cancer, *NMIBC* non-muscle invasive bladder cancer, *AML* acute myeloid leukemia

#### PD-1 and PD-L1 blockade

**Fig. 2:**
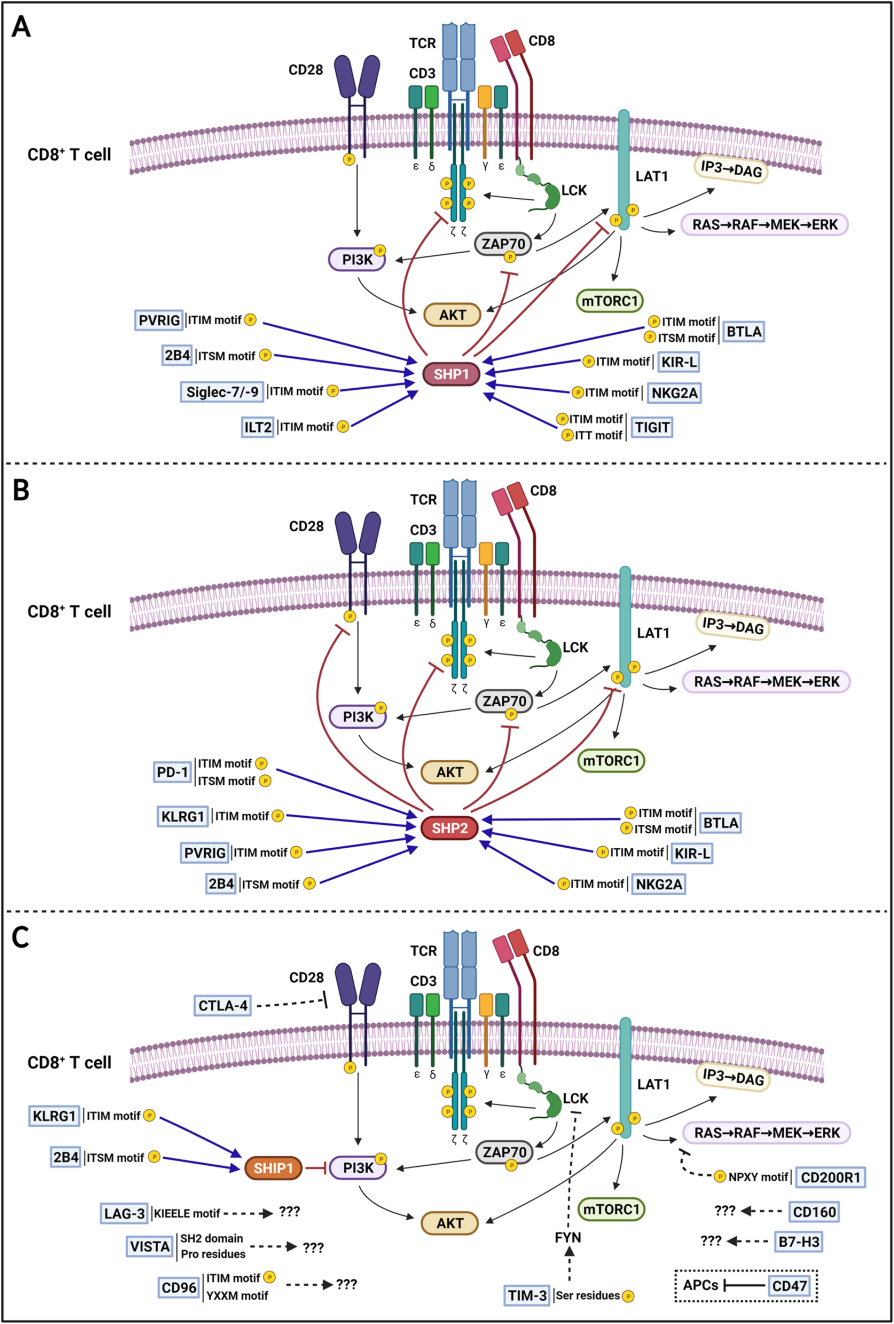
Immune checkpoint downstream inhibitory signaling in CD8^+^ T cells. Immune checkpoint pathways initiated after binding of ligands to their respective IC receptors (blue boxes) interfere with TCR signaling by a variety of mechanisms. ICs have inhibitory motifs in their cytoplasmic tail that can recruit (blue arrows) protein tyrosine phosphatases SHP1 and/or SHP2, which are responsible for dephosphorylating (red inhibitory arrows) TCR downstream signaling proteins. This is the case for PVRIG, 2B4, Siglec-7/−9, ILT2, BTLA, KIR-L, NKG2A, TIGIT, PD-1, and KLRG1. However, some ICs, such as CTLA-4, TIM-3, CD47, and CD200R1, present alternative downstream mechanisms, while other IC downstream signaling, such as that involving LAG-3, VISTA, CD96, CD160, and B7-H3, remains to be fully elucidated. Schematic representation of (**A**) SHP1-dependent inhibition of TCR signaling, (**B**) SHP2-dependent inhibition of TCR signaling, and (**C**) non-dependent SHP1 and SHP2 inhibition of TCR signaling. Dotted lines indicate indirect mechanisms (created with BioRender.com)

PD-1 promotes inhibitory signals upon binding to its ligands, programmed death-ligand 1 (PD-L1) and programmed cell death 1 ligand 2 (PD-L2), in T cells [12]. PD-1 is expressed in distinct immune cell populations, including those of B, Treg, and myeloid cells. While PD-L1 may be expressed in hematopoietic and non-hematopoietic cells, physiological expression of PD-L2 is restricted to DCs, macrophages, and B cells. Notably, PD-L1 and PD-L2 expression is upregulated in many tumor types and is usually associated with poor patient outcome. PD-1 regulates activated T cell function in the later stages of the immune response in peripheral tissues [15]. Activated T cells induce PD-1 expression, and its signaling, upon binding to PD-L1/−L2, decreases T cell proliferation and cytokine secretion. PD-1 cytoplasmic tail contains classic inhibitory motifs ITIM and ITSM which recruit SHP-2 phosphatases resulting in reduced TCR downstream phosphorylation and signaling [20] (Fig. 2B). High PD-1 levels, concomitantly with other ICs, can be detected in TILs and in association with an exhausted phenotype [21]. PD-1 signaling in Treg cells enhances their proliferative and suppressive functions [5]. In NK cells, PD-1 expression has only been identified under pathological conditions [22]. PD-1 is upregulated in tumor-infiltrating NK cells and PD-1^+^ NK cells are known to be functionally exhausted [23]. Although PD-1 downstream mechanisms leading to NK exhaustion have not been completely elucidated, SHP-2 recruitment might participate in the process, given its role damping NK cell function [24]. An interaction between PD-L1 and CD80 expressed in T cells was also characterized, which led to inhibition of T cell function in vitro [25]. However, other studies suggest that the PD-L1 interaction with CD80 could cause T cell expansion without promoting exhaustion. Specifically, it has been reported that in bone marrow transplantations, the interaction of CD80 from donor CD8^+^ T cells with PD-L1 in lymphoid tissues from recipient patients, promotes T cell expansion, resulting in increased graft-versus-leukemia activity [26]. According to these findings, the blockade of PD-L1 signaling could reduce the antitumoral activity of T cells in specific tissue environments.

PD-1 signaling blockade increases antitumor immunity and decreases tumor growth. Specifically, PD-L1 transgenic expression in tumor cells enhances tumorigenesis, which can be reversed with anti-PD-L1 antibodies. PD-L1 mAb blockade enhances DC-mediated T cell activation and antitumor function. Tumor-infiltrating exhausted NK cells express PD-1 and its blockade partially restores NK antitumoral activity [23]. Although PD-1 signaling in Treg cells enhances immunosuppressive functions, in vivo PD-1 blockade in Treg cells leads to different tumor-dependent responses [27]. Moreover, a complete anti-PD-1 antitumoral effect requires DC stimulation and function [28]. A bispecific anti-PD-1/PD-L1 antibody gave rise to greater antitumoral efficacy relative to monospecific therapies in a high-grade serous ovarian cancer model by inducing a superior cytotoxicity in both T and NK cells [29]. This suggests that PD-L1 could be involved in interactions other than those binding to PD-1. PD-1 blockade alongside other ICs has shown itself to increase T cell response [30] and to bestow therapeutic benefits, which will be further discussed in the corresponding IC blockade section.

Inhibition of the PD-1/PD-L1 axis is the most commonly applied IC blockade therapy. Nivolumab, pembrolizumab, and cemiplimab are PD-1-blocking mAbs that have proven therapeutic efficacy in treating patients suffering from different tumor types (Table 2). Multiple anti-PD-1 antibodies, including these three, are being tested either alone (Table S1) or in combination with other anti-IC antibodies in multiple cancers (Table 4). The anti-PD-L1 antibodies, atezolizumab, durvalumab, and avelumab, also have a therapeutic value in treating certain tumor types and have been approved for clinical use (Table 2). These three antibodies, among others, are being tested as treatments for a wide range of solid tumors in clinical trials, either as monotherapy (Table S1) or in combination with other IC blockade antibodies (Table 4).

### IC inhibitors under clinical development

#### LAG-3 blockade

The coinhibitory receptor lymphocyte activation gene-3 (LAG-3) is an IC receptor expressed by CD8^+^ T cells and NK cells that regulates peripheral tolerance. LAG-3 binds to HLA class II as well as to fibrinogen-like protein 1 (FGL-1), Galectin-3 (Gal-3), and LSECTin. LAG-3 ligands are expressed by tumor cells, and, notably, FGL-1 participates in immune evasion mechanisms that reduce the T cell response [31]. Mice deficient in LAG-3 have altered T cell proliferation. LAG-3 is expressed in Treg cells and its blockade disrupts Treg suppressor functions [32]. LAG-3 is also expressed in NK cells, but its function is not yet fully understood. Blockade of LAG-3 in human NK cells does not induce NK cytotoxicity against different tumor types [18]. Recent findings indicate that LAG-3 blockade in vitro increases cytokine production by NK cells without affecting their cytotoxicity [33]. The mechanism of action of LAG-3 remains largely unknown. LAG-3 cytoplasmic tail does not contain classical inhibitory motifs but presents a KIEELE motif that may mediate LAG-3 inhibitory functions [34] (Fig. 2C).

Anti-LAG-3 mAb administration was found to increase the proliferation and effector function of CD8^+^ T cells and delay tumor growth in a prostate cancer mouse model. LAG-3 is co-expressed with PD-1 in CD4^+^ and CD8^+^ TILs in many mouse and human tumors [35]. Simultaneous blockade of PD-1 and LAG-3 synergizes to enhance anti-tumor CTLs activity and reduces tumor growth in a colon adenocarcinoma model [36], a chronic lymphocytic leukemia (CLL) model [37], and a malignant pleural mesothelioma model [38]. LAG-3 expression has been linked to a stronger suppressive function of Treg cells [39]. The role of NK cells in LAG-3 blockade therapy response, and the possible contribution of NK cells to the observed antitumoral effects remain largely unknown. A unique study determined that treatment of the 4T1 mouse model of metastatic breast cancer with IL-12, combined with anti-LAG-3 or anti-PD-1, recovered NK cell cytotoxicity and proliferation, which resulted in a reduced presence of pulmonary metastases [40]. LAG-3 blockade is currently being investigated in clinical trials, either as monotherapy or combined with the inhibition of other ICs, to treat multiple tumor types (Table 4).

#### TIM-3 blockade

T-cell immunoglobulin and mucin-domain containing-3 (TIM-3) acts as a coinhibitory receptor of T cells and is also expressed in Tregs, NK cells, DCs, and macrophages. TIM-3 expression is known to be increased in exhausted TILs [41]. The first TIM-3 ligand to be described was Galectin-9 (Gal-9), but TIM-3 also binds to phosphatidylserine (PS), carcinoembryonic antigen-related cell adhesion molecule 1 (CEACAM1), and high-mobility group protein 1 (HMGB1). The expression of TIM-3 ligands is upregulated by APCs, endothelial cells, and neutrophils, among other immune cells, and has been linked to carcinogenesis and tumor progression. Gal-9 interaction with TIM-3 negatively regulates T-helper function and can induce T-cell death [42]. TIM-3 blockade and gene depletion downregulate Th1 cell function and increase CTL proliferation and cytokine production [31]. TIM-3^+^ Treg cells increase suppressor functions in vitro [43]. Blockade of TIM-3 reverses the exhausted phenotype of CTLs from patients with advanced tumors. TIM-3 interaction with its ligands leads to the phosphorylation of conserved tyrosine residues in its cytoplasmic tail by the tyrosine ITK [44]. Phosphorylation of the TIM-3 cytoplasmic tail leads to the release of BAT3 protein and the recruitment of the tyrosine kinase FYN, ultimately resulting in TCR downstream kinase LCK inhibition [45] (Fig. 2C). In NK cells, TIM-3 acts as a negative regulator of NK effector functions. Its expression is upregulated in cancer, and has been associated with NK exhaustion [18]. Additionally, TIM-3 ligand Gal-9 can also interact with PD-1 expressed by T cells, which dampens the CD4^+^ and CD8^+^ T cell response [42, 46]. Specifically, Gal-9 interacts with PD-1 through glycans without affecting the PD-1/PD-L1 interaction. Likewise, the Gal-9/PD-1 interaction enables the formation of PD-1 and TIM-3 heterodimers, which promotes T cell exhaustion but dampens Gal-9/TIM-3-induced T cell apoptosis [47]. It is of note that, human Treg cells express high levels of Gal-9, which induces Treg death upon interacting with TIM-3 expressed by tumor resident cells [47]. Hence, anti-Gal-9 therapy might promote Treg function, thereby limiting the antitumoral function of CD8^+^ T cells. In this sense, the combination of anti-Gal-9 with Treg cell treatment depletion promotes synergistic antitumor activity in a breast tumor model by activating specific subsets of TILs [47].

In a TIM-3-overexpressing mouse model, anti-TIM-3 antibody reduces tumor growth by restoring T-cell activity [48]. Concomitant blockade of PD-1 and TIM-3 further improves T cell anti-tumor function and reduces tumor growth more effectively. Simultaneously targeting TIM-3 and PD-1 increases LAG-3 expression in TILs, suggesting a cross-regulation between IC receptors, and triple blockade of TIM-3, PD-1, and LAG-3 results in reduced tumor growth in a colon adenocarcinoma model [49]. Simultaneous blockade of TIM-3 and PD-L1 significantly reduces tumor growth in orthotopic models of HNSCC [50] whereas treatment with anti-TIM-3 concurrently with anti-PD-1 causes greater regression of murine glioma than is produced by a single checkpoint blockade [51]. TIM-3 blockade reverses exhausted NK cells isolated from lung adenocarcinoma patients [52], and advanced MHC class I-deficient tumors treated with IL-21 combined with anti-TIM-3 and anti-PD-1 reduce tumor progression by enhancing NK cell antitumoral immunity [53]. Hence, TIM-3 blockade also boosts NK cytotoxic activity in specific tumor settings. The presence of TIM-3^+^ Treg cells is associated with poor prognosis in lung cancer, and pharmacological blockade of TIM-3 reduces the suppressive function of intratumoral Treg cells [41]. In vitro experiments indicated that TIM-3 signaling in DCs blocks DC activation and maturation [54]. A recent study concluded that the accumulation of TIM-3^+^ CD4^+^ T cells in tumoral regions favors TIM-3-mediated immunosuppressive functions in hepatocellular carcinoma (HCC) patients. Depletion of CD4^+^ T cells abrogates the antitumoral effects of anti-TIM-3 therapy, indicating that CD4^+^ T cells might be responsible for TIM-3-mediated immunosuppression [55]. The response of TIM-3 blockade-based therapy is currently being analyzed in clinical trials as monotherapy or in combination with anti-PD-1 antibodies (Table 4).

#### TIGIT blockade

The T cell immunoglobulin and ITIM domain (TIGIT) is a member of the immunoglobulin superfamily of paired receptors expressed by T cells and NK cells that interact with ligands of the nectin and nectin-like family. TIGIT acts as an inhibitor receptor when binding to its ligands PVR, nectin-2, and nectin-3 (CD113), all of which are expressed in APCs and a variety of non-hematopoietic cells, including tumor cells [56]. Specifically, TIGIT interacts with PVR with a higher affinity than with nectin-2, while the nectin-3/TIGIT interaction has a weak binding affinity. In addition, DNAM-1 is another co-stimulatory receptor expressed by NK and T cells that competes with TIGIT to bind PVR and nectin-2, which enables it to regulate T cell inhibition precisely [57]. In NK cells, TIGIT signaling reduces NK cytotoxicity and cytokine release, while in T cells it reduces T cell activation, proliferation, and effector functions. Specifically, the TIGIT intracellular cytoplasmic tail contains an ITIM and an ITT-like domain that recruit SHP-1 phosphatases, leading to the blockade of PI3K and MAPK pathways in NK cells [58], and decreased TCR downstream signaling [59] (Fig. 2A). TIGIT^+^ Treg cells have greater suppressive capacities in vitro and selectively suppress the Th1 pro-inflammatory response in vivo [31]. TIGIT interaction with PRV expressed by DCs forces the DCs to be tolerogenic by increasing their production of immunosuppressive IL-10 cytokine [60].

TIGIT ligands are expressed by cancer cells and exhausted TIGIT^+^ T and NK cells are detected in various human cancers. Antibody blockade of TIGIT enhances antitumor CD8^+^ T cell response and prompts tumor regression in a colorectal cancer (CRC) mouse model. TIGIT dual blockade with PD-1, PD-L1, or TIM-3 has a synergistic action that produces enhanced CD8^+^ T cell activity and tumor regression in a colorectal carcinoma model [61]. Dual blockade of PD-1 and TIGIT enhances antitumor response and increases survival in a mouse glioblastoma model [62]. Conditional TIGIT KO in Treg cells reduces tumor growth in a melanoma mouse model, proving that the TIGIT blockade effect is also mediated by Treg cells [31]. Recently, it was demonstrated that TIGIT blockade elicits NK-mediated antitumoral immunity in tumor-bearing mouse models [63]. Antibodies targeting TIGIT are currently being investigated in clinical trials to treat patients with different tumor types (Table 4).

#### PVRIG blockade

PVRIG, also known as CD112R, is another member of the immunoglobulin superfamily of paired receptors. It has recently been identified in human T and NK cells. PVRIG binds to nectin-2 but does not recognize PVR. In T cells, PVRIG signaling inhibits the TCR-mediated signal dampening T cell response by recruiting SHP-1 and SHP-2 phosphatases to its ITIM-like domains [64] (Fig. 2B). Human NK cells expressing PVRIG present reduced proliferation and cytokine release [56]. Recent results suggest that PVRIG expression is reduced during human NK-cell activation [65], while exhausted tumor-infiltrating NK cells express high levels of PVRIG [66]. Until now, expression of PVRIG has not been described in myeloid immune cell populations.

Targeting PVRIG with antibodies promoted T cell expansion in vitro, which was further increased by simultaneous blockade with TIGIT [64]. PVRIG blockade enhances in vitro NK cell antitumoral activity and increases IFN-γ release [67]. In AML patients, anti-PVRIG therapy promotes NK-cell cytotoxicity against AML blasts [65]. In mouse models of cancer, TILs show high PVRIG levels [68]. Ex vivo PVRIG blockade of patient-derived T cells upregulates T-cell function, an effect that is further enhanced when combined with anti-TIGIT or anti-PD-1 treatments [56]. Anti-PVRIG treatment delays tumor growth and prolongs survival of tumor-bearing mice by reversing exhaustion of NK and CD8^+^ T cells of solid tumors. Dual blockade of PVRIG and PD-L1 enhances the antitumoral effects in comparison to single-blockade therapies in solid tumors [66, 68]. Notably, PVRIG^−/−^ mice exhibit reduced tumor growth in a CD8^+^ T cell-dependent manner, an effect that is synergistically enhanced by PD-L1 blockade. Finally, PVRIG is co-expressed with PD-1 and TIGIT in activated T cells and combinatory dual blockade of PVRIG with PD-1 or TIGIT additionally promoted T-cell activity [69]. An anti-PVRIG mAb is currently being tested in a clinical trial in patients with advanced solid tumors (Table 4).

#### KIR-L blockade

Killer immunoglobulin-like (KIRs) are a family of receptors that regulate self-tolerance and effector functions of NK cells by binding to classic HLA class I allotypes (HLA-A, HLA-B, and HLA-C). These receptors allow the identification and elimination of cells that fail to express a sufficient level of HLA, like many tumor cells, in a process called missing self-recognition. KIR receptors with a long cytoplasmic tail (KIR-L) mainly present NK coinhibitory capabilities, while short-tailed KIR receptors (KIR-S) enhance NK function [70]. The KIR-L cytoplasmic tail contains ITIM motifs that become phosphorylated upon binding with its ligand, and recruit tyrosine phosphatases such as SHP-1 and SHP-2, resulting in NK-cell inhibition [71] (Fig. 2A and B). KIRs were initially characterized as NK cell receptors, but they are also expressed in CD8^+^ T-cell subsets. It has been proposed that KIR expression can be sustained by TCR engagement, and may participate in T-cell tolerance to self-antigens [72].

Initial evidence that the blockade of KIR signaling could be beneficial in treating cancer came with the observation that acute myeloid leukemia (AML) patients did not experience recurrence within 5 years if they received a bone marrow transplantation from a donor who presented NK with KIRs that mismatched the interaction with the HLA of the patients [73]. Thus, the lack of recognition of HLA class I molecules by KIRs enhances NK cell cytotoxic activity, causing the elimination of malignant cells. Additionally, tumor-infiltrating NK cells have a high level of expression of inhibitory KIRs that are correlated with poor clinical outcome in non-small-cell lung cancer (NSCLC) [74]. A unique mAb targeting KIR2DL1, KIR2DL2, and KIR2DL3 receptors increases NK-cell cytotoxicity against autologous AML blasts and multiple myeloma cells [18]. Hence, KIR blockade appears to enhance NK cell function in the tumors. Different KIR inhibitors are being tested in clinical trials as single agents or combined with anti-PD-1 or anti-CTLA-4 antibodies (Table 4).

#### NKG2A blockade

The natural killer group 2A (NKG2A) is a member of the NKG2 receptor group, which dimerizes with CD94 to bind to non-classic HLA-E class I molecules, which are ubiquitously expressed. Upon binding to its ligand, CD94/NKG2A signaling downregulates NK cell-mediated cytotoxicity. Tumor-infiltrating NK cells from patients with liver cancer express high levels of NKG2A and are functionally exhausted [75]. NKG2A and CD94 expression is restricted to a subset of CD8^+^ T cells [76] and its expression in TILs generates negative regulatory signals. Mechanistically, NKG2A contains cytoplasmic ITIM motifs responsible for its inhibitory functions by recruiting the phosphatases SHP-1 and SHP-2 [77] (Fig. 2A and B).

Combined blockade of NKG2A and PD-1 or PD-L1 synergizes to reduce tumor growth in a mouse model of B-cell lymphoma by promoting NK and CD8^+^ T cell cytotoxic activity [78]. Anti-NKG2A blockade enhances NKG2A^+^ NK cells’ cytotoxic function, eliminating human leukemia cells engrafted in mice [79]. Monalizumab is a humanized mAb that specifically blocks NKG2A and enhances NK and T cell effector function and promotes anti-tumor immunity [78]. Monalizumab is under analysis in clinical trials in which it is used either alone or in combination with anti-PD-L1 antibodies to treat a variety of tumor types (Table 4).

#### CD200 blockade

CD200 is a cell-surface glycoprotein that binds to the coinhibitory receptor CD200R1 expressed in subsets of NK, T, B, and myeloid cells. CD200 expression is detected in various human tissues such as endothelium, central nervous system, retina, and in activated DCs, T, and B cells. It is of note that the interaction between CD200 and CD200R1 occurs between different cells expressing ligand and receptor (in *trans*), while the interaction in the same cell (in *cis*) is remote due to steric constraints [80]. CD200R1 signaling in NK cells inhibits their cytotoxic activity and cytokine release [18]. CD200R1 contains a NPXY motif in its cytoplasmic tail that when phosphorylated inhibits the Ras/MAPK signaling [81] (Fig. 2C). A direct effect of CD200R1 signaling on T cell activity is less clear. While one study in CD200R KO mice suggested greater T-cell cytotoxicity, another showed a normal T-cell response with a lack of CD200R1 signaling [82]. In DCs and macrophages, CD200R1 signaling reduces pro-inflammatory cytokine production, leading to a tolerogenic state [82]. CD200 may have indirect inhibitory effects on T-cell activity by modifying the cytokine landscape rather than a direct cell-intrinsic inhibitory signal. No direct effect of CD200R1 signaling in other CD200R-expressing immune populations has been reported.

CD200^−/−^ mice displayed reduced rate of skin carcinogenesis [82]. CD200^+^ leukemic cells from AML patients reduced NK cytotoxic activity relative to CD200^−^ leukemic cells and NK function was recovered with anti-CD200 mAb [83]. CD200 blockade increased antitumoral response in a mammary carcinoma mouse model [84]. Finally, CD200 blockade stimulated T cell antitumoral response in CLL patients [85]. Samalizumab, an antibody that targets CD200, is being tested in clinical trials (Table 4). No anti-CD200R1 antibodies have been evaluated in preclinical or clinical contexts as antitumoral therapy.

#### CD47 blockade

CD47, originally called integrin-associated protein (IAP), is a transmembrane glycoprotein of the immunoglobulin superfamily that is expressed ubiquitously by human cells. When CD47 binds to its ligands, the signal regulatory protein alpha (SIRPα) and thrombospondin-1 (TSP1) can induce cell activation or apoptosis, depending on the cellular context. CD47 activation in immune cells has been linked to tumor immune evasion, decreased antigen-presentation cell function, and impaired effector functions of NK and T cells [86]. In vivo experiments have shown that CD47 signaling inhibits NK and T cell cytotoxicity indirectly through impaired APC functions [87, 88]. Notably, the best characterized function of CD47 serves as an antiphagocytic signal for macrophages upon binding to SIRPα [86].

CD47 blockade indirectly enhances anti-tumor cytotoxicity by stimulating macrophage phagocytosis and antigen presentation by APCs, which enhances CD8^+^ T cell cytotoxicity. In this sense, the antitumoral effects of anti-CD47 treatment are abrogated in T cell-deficient mouse models [88]. Head and neck squamous cell carcinoma (HNSCC) cells with a high level of expression of CD47 show lower NK cytotoxicity, which is reversed upon anti-CD47 treatment [87]. Another strategy that has been developed involves the direct blockage of the SIRPα ligand. An anti-SIRPα antibody reduced tumor growth in an NK- and T cell-dependent manner [86]. A recent study in a mouse model of breast cancer demonstrated that the simultaneous blockade of CD47 and PD-L1 inhibits tumor growth by enhancing T- and NK-cell activity [89]. The targeting of CD47 is being tested in clinical trials for the treatment of various tumor types (Table 4).

#### BTLA blockade

B and T cell lymphocyte attenuator (BTLA) is a coinhibitory receptor expressed in T cells, DCs, and B cells, but not in NK cells. BTLA binds to the herpesvirus entry mediator (HVEM) protein, which is expressed by B cells, DCs, and T cells. In naïve T cells, BTLA-HVEM interaction in *cis* inhibits T-cell activation, ensuring a quiescent state [90]. At the signaling level, BTLA cytoplasmic tail contains ITIM and ITSM motifs responsible for recruiting the tyrosine phosphatases SHP-1 and SHP-2 which reduces TCR downstream phosphorylation [91] (Fig. 2A and B). TILs exhibit upregulated expression of BTLA, less proliferation, and less extensive cytokine production when they interact with HVEM expressed by cancer cells. Indeed, BTLA and PD-1 co-expression is detected in exhausted TILs from patients with advanced melanoma [92]. Finally, DCs expressing BTLA, when interacting with HVEM, promote the differentiation of peripheral Treg cells, and induce antigen tolerance [93].

Anti-BTLA therapy combined with chemotherapy reduces tumor growth, and increases the survival of tumor-bearing mice [94]. Recent proteomic studies have revealed a rationale for simultaneously blocking PD-1 and BTLA in order to increase the T-cell antitumoral response [95]. In this regard, BTLA blockade upon anti-PD-1 and anti-TIM-3 treatment further increases CD8^+^ T cell proliferation [92]. Interestingly, in ovarian carcinomas BTLA expression was mainly identified in B lymphocytes rather than T or NK cells, and the BTLA blockade antitumoral effect was caused by inhibiting a specific subset of B lymphocytes rather than stimulating T or NK cell function [94]. Two mAbs targeting BTLA are currently being studied in early-phase clinical trials (Table 4).

#### VISTA blockade

V-domain Ig suppressor of T cell activation (VISTA), also known as programmed death-1 homolog (PD-1H), is an IC expressed by CTLs, Treg cells, DCs, macrophages, and neutrophils, but not NK cells. VISTA has been considered to be a ligand or a receptor in different studies. Here, we will consider it to be a receptor, since the interaction with its ligand leads to VISTA intracellular downstream signaling. VISTA is a functionally pH-selective receptor and interacts with the P-selectin glycoprotein ligand-1 (PSGL-1) under acid pH conditions [96]. PSGL-1 is expressed by different cell populations as T, B, endothelial cells, DCs, macrophages, monocytes, and neutrophils, and participates in leukocyte homing processes. VISTA signaling suppresses T-cell activation and proliferation in in vivo experiments [97]. The VISTA cytoplasmic tail contains conserved proline residues and an SH2 domain that may be responsible for its inhibitory functions [98] (Fig. 2C).

Anti-VISTA antibodies reduce tumor growth and increase T-cell tumor infiltration and effector functions in preclinical models. VISTA blockade decreases the suppressive functions of Treg cells, reduces the intratumoral presence of MDSCs, and increases tumor infiltration of activated DCs [99]. Dual blockade of PD-1 and VISTA synergistically enhance the antitumor T-cell response in various mouse models [100]. Finally, given that VISTA binds its ligand, PSGL-1, under low pH conditions, the possibility of using engineered pH-selective antibodies that bind its epitope in specific pH environments has arisen. The synergy between an acidic pH-selective VISTA-blocking antibody and an anti-PD-1 antibody has been shown to reduce tumor growth in a mouse model of colon adenocarcinoma [96]. VISTA blockade has been translated to the clinical milieu for testing as an anticancer therapy (Table 4).

#### B7-H3 blockade

The B7 homolog 3 protein (B7-H3) is a B7 family molecule that functions as a ligand, but its receptor remains to be discovered. T lymphocytes and NK cells respond to B7-H3, suggesting that the B7-H3 receptor is expressed in these immune cell types [18]. B7-H3 has been reported as playing a variety of roles in T cells. B7-H3 signaling increases T-cell proliferation, cytokine release, and enhances antitumor T cell activity in cancer mouse models [101]. Conversely, B7-H3 signaling in T cells is also able to inhibit them by blocking TCR signaling [102]. These contradictory effects might be due to specific TME features interfering with T-cell function. B7-H3 functions as an inhibitory ligand for NK cells, and anti-B7-H3 antibodies enhance NK-cell cytotoxicity in vitro. Various human cancer cells upregulate B7-H3 expression, and are related to impaired T-cell function, suppressed NK cytolytic activity, and tumor immune evasion [18].

Anti-B7-H3 treatment reduces tumor growth in cancer mouse models that express B7-H3. B7-H3 KO glioma-initiating cells show less invasiveness and higher susceptibility to NK cell-mediated cytolysis [103]. Other preclinical studies have indicated that higher levels of B7-H3 may be beneficial in the T-cell-mediated anti-tumor response against mastocytoma. A synergistic antitumoral response between anti-B7-H3 mAbs and chemotherapy has been observed in several preclinical models [104]. Thus, blockade of B7-H3 may be beneficial, boosting T and NK cell effector functions in specific tumor types and cellular contexts. Finally, the combination of anti-B7-H3 and anti-PD-L1 treatment promotes a synergistic antitumor response relative to single-agent blockades in a mouse model of NSCLC [105]. Antibodies blocking B7-H3 are being tested in clinical trials for the treatment of several tumor types (Table 4).

### IC inhibitors under preclinical investigation

#### CD96 blockade

CD96, also known as T cell activation increased late expression protein (TACTILE), is a member of the immunoglobulin superfamily of paired receptors which contains intracellular inhibitory and activating motifs. The cytoplasmic tail of CD96 contains an inhibitory motif ITIM and a YXXM motif that is thought to mediate activating or inhibitory functions depending on the cell type [106] (Fig. 2C). CD96 binds to PVR and nectin-1 (CD111) and is expressed in T and NK cells. It has been reported that CD96 signaling in NK cells negatively controls cytokine release [56]. CD96’s role in CD8^+^ T cells is controversial. Cross-linking CD96 in CD8^+^ T cells induces proliferation and effector cytokine production [107]. However, recent findings indicate that CD96 blockade inhibits primary tumor growth in various tumor mouse models, an effect that is dependent on CD8^+^ T cell activity [108]. Further studies are needed to determine the role of CD96 in T cells under pathological conditions.

The presence of exhausted CD96^+^ NK cells is associated with poor clinical prognosis in HCC patients. CD96 blockade increases NK cell-mediated lysis and synergizes with an anti-TIGIT antibody to produce an enhanced antitumoral effect. CD96^−/−^ mice injected with B16 melanoma cells develop fewer lung metastases, this reduction being dependent on NK cells. Another study reported decreased metastasis development after anti-CD96 treatment in several preclinical models [18]. Dual blockade of CD96 with PD-1, PD-L1, TIGIT, or CTLA-4 increases antitumor response, and triple blockade of CD96, PD-1 and TIGIT yields the highest level of antitumoral immunity in various mouse tumor models [108]. To date, antibodies blocking CD96 have not been evaluated in clinical trials.

#### ILT2 blockade

The immunoglobulin-like transcript-2 (ILT2), also known as leukocyte immunoglobulin-like receptor-1 (LIR1), is a coinhibitory receptor expressed by NK cells, subsets of T, B cells, and DCs. Non-classic HLA-G class I molecules are ILT2 ligands. HLA-G maintains fetal-maternal immune tolerance, and is expressed in adult tissues in cancer. ILT2 signaling inhibits NK-cell effector functions by decreasing cytotoxicity and IFN-γ release. ILT2 interaction with HLA-G inhibits T-cell proliferation and related cytolysis by recruiting SHP-1 phosphatase to its cytoplasmic ITIM domain [109] (Fig. 2A). In DCs, ILT2 signaling induces the development of tolerogenic DCs. Furthermore, exposure of HLA-G molecules to DCs induces anergy in CD4^+^ and CD8^+^ T cells, and impairs NK cytolytic functions [110].

Simultaneous blockade of ILT2 and NKG2A increases the cytotoxicity of NK cells to acute myelogenous leukemia and acute lymphoblastic leukemia human blasts. Moreover, ILT2 blockade increases NK-cell cytotoxicity, leading to the elimination of malignant cells from CLL patients [111]. At present, no ILT2 blockade antibodies have been reported in clinical trials.

#### Siglec-7 and Siglec-9 blockade

Sialic acid-binding immunoglobulin type lectins (Siglecs) are cell-surface receptors of the I-type lectin family that bind sialic acid-containing glycans present on glycoproteins and glycolipids. Siglec-7 and Siglec-9 are expressed by human NK cells, negatively regulating NK effector function [18]. CD8^+^ T cell subsets expressing Siglec-7 and Siglec-9 receptors present reduced activity. Specifically, Siglec-7 and Siglec-9 contain cytosolic inhibitory ITIM motifs responsible for recruiting the phosphatase SHP-1 [112] (Fig. 2A). In monocytes, Siglec-7 signaling induces the release of pro-inflammatory molecules upon pathogen recognition [113]. Siglec-9 signaling in macrophages reduces LPS-induced CCR7 expression, revealing a role in modulating innate immunity [114], while Siglec-9 ligand interaction with immature DCs reduces LPS-induced IL-12 release [115]. Finally, the presence of molecules containing sialic acid modifications, including hypersialylation and xenosialylation, has been linked to tumor progression [116].

Siglec-9 is co-expressed with PD-1 in TILs from patients with various cancer types [117]. Targeting Siglec signaling pathways enhances the antitumoral response, while genetically modified mice expressing Siglec-9 in T cells show accelerated growth of CRC tumors [117]. Isolated NK cells from cancer patients with upregulated Siglec-9 expression are less cytotoxic. Anti-Siglec-7 and anti-Siglec-9 antibodies strengthen the effector function of NK cells against cancer cell lines expressing Siglec-7 or Siglec-9 ligands [118]. At present, there are no reports of Siglec-7 or Siglec-9 antibodies being assessed in clinical trials.

#### KLRG1 blockade

KLRG1 is a coinhibitory receptor expressed exclusively in NK and T cells that binds to E-cadherin and N-cadherin. KLRG1 signaling inhibits NK-associated cytotoxicity and reduces IFN-γ production [119]. In T cells, KLRG1 signaling inhibits cell proliferation and cytotoxic activity [120]. Upon binding to its ligands, KLRG1 recruits SHIP-1 and SHP-2 phosphatases but not SHP-1 to its cytoplasmic ITIM domains resulting in interfered TCR signaling [121] (Fig. 2B and C).

Although KLRG1 KO mice do not display increased NK or T cell cytotoxic functions, pharmacological targeting of KLRG1 increases the effector functions of NK and T cells [122]. Antibody blockade of KLRG1 restores NK-cell cytotoxicity of genetically engineered KLRG1-expressing NK cells in vitro [123]. Administration of anti-KLRG1 antibody does not modify primary tumor growth but has been shown to reduce lung metastases in breast cancer mouse models [124]. Notably, dual blockade of KLRG1 and PD-1 has been shown to decrease primary tumor growth synergistically in melanoma and CRC mouse models [124]. No clinical trials involving anti-KLRG1 antibodies have so far been reported.

#### 2B4 Blockade

2B4, also known as CD244, is a coinhibitory receptor expressed by myeloid cells, NK cells, and a subset of CD8^+^ T cells. CD48, which is 2B4 ligand, is ubiquitously expressed by hematopoietic cells and upregulated in hematological malignancies [125, 126]. 2B4 receptors contains inhibitory ITSM motifs responsible of recruiting the tyrosine phosphatases SHP-1, SHP-2 and SHIP-1 [127] (Fig. 2). In human NK cell precursors, 2B4 presents inhibitory functions, whereas in mature NK cells, 2B4 signaling enhances their cytotoxic activity [128]. NK cells and CD8^+^ T cells expressing 2B4 from human cancer patients present an exhausted phenotype that can be reversed by the blockade of the 2B4-CD48 interaction [129]. Furthermore, 2B4 expression levels in tumor-infiltrating DCs and MDSCs are correlated with tumor cell PD-L1 expression and MDSC production of immunosuppressive molecules [130].

In vitro blockade of 2B4 with mAbs increases NK-cell and T-cell functions in 2B4^+^-exhausted NK and T cells [129]. A 2B4 KO mouse model revealed increased rejection of engrafted melanoma cells [131]. In addition, 2B4 KO mice presented impaired HNSCC growth in a preclinical model. Anti-2B4 mAb treatment phenocopies 2B4 KO mice, inhibiting tumor growth and increasing the presence of TILs [130]. No 2B4 blockade therapies have so far been tested in clinical trials.

### Current challenges: improving efficacy without increasing adverse events

Although immunotherapy based on IC blockade has produced promising results in a fraction of patients, a large number of patients do not benefit from the existing approved drugs (Table 3). Tumor-infiltrating immune cells present in the TME play a fundamental role in therapy response. Tumors that are not likely to generate a robust immune response have been classified as poorly immunogenic or cold tumors. These tumors have low quantities of immune infiltrate [1]. On the other hand, hot tumors have high levels of T cell infiltration and are highly immunogenic. Targeting the TME to transform cold tumors into hot tumors before IC blockade therapies is being investigated as a strategy to increase the responsiveness to these therapies. The approaches promoting immunogenicity of cold tumors include enhancing antigen presentation by DCs, reducing the presence and function of immunosuppressive cells, and delivering immunomodulatory factors to boost inflammation [132].

Combining IC blockade drugs with other immunotherapeutic agents also produces promising enhanced antitumoral responses. Chimeric antigen receptor (CAR) T cells are engineered autologous T cells with an artificial TCR that recognize a specific antigen in an MHC-independent manner. CAR T cells achieve long-lasting responses in hematological malignancies, whereas the clinical activity observed so far in solid tumors has been more modest. This may occur because the immunosuppressive TME that present solid tumors can lead to CAR T cell exhaustion [133]. Hence, combining CAR T cells with anti-IC drugs and/or drugs targeting the immunosuppressive TME may ideally produce a synergistic effect, unleashing CAR T cell activity against tumoral cells. Combining anti-PD-1 antibodies with adoptive transfer of CAR T cells might overcome PD-1 dependent T cell exhaustion, thereby improving single-treatment responses [134, 135]. Another interesting strategy to overcome the exhaustion of CAR T cells in solid tumors is to use PD-1 KO CAR T cells, which have enhanced cytotoxic activity [136]. Given the crucial role of APCs in priming antigen-specific T cell immunity, the modulation of APC function has also been used as an antitumoral strategy. In this regard, several DC-based immunotherapies are being clinically studied, such as treatment with immunostimulatory molecules that promote DC activity or vaccine administration of tumor-associated antigens (TAAs) that can be processed and presented by endogenous DCs [137]. The use of DC vaccines consisting of ex vivo-amplified autologous DCs, tumor antigen load and reinfusion to patients, has also proven clinical benefits [137]. Combining dendritic cell vaccines with anti-PD-1 therapies may boost efficacy by improving T cell antitumoral function in mouse models [138, 139]. In addition, DC vaccination can overcome IC blockade-resistant murine lung cancers by eliciting an antitumoral response [140]. Hence, the appropriate combination of immunotherapeutic agents is a promising strategy for treating single-agent-resistant tumors. In addition, chemotherapy and radiotherapy are standard cancer therapies used in the clinic setting that produce immunostimulatory effects and that can be combined with IC blockade therapies to increase antitumoral immunity [141, 142].

Despite the positive results of IC blockade therapies as single agents or in combination, patients treated with IC inhibitors may suffer from secondary autoimmune events, also known as immune-related adverse events (irAEs) [143]. Immune cell exhaustion, promoted by the activation of IC pathways, among other mechanisms, prevents overactivation of effector immune cells and preserves normal homeostasis and self-tolerance. Genetic ablation of ICs leads to the development of autoimmune diseases in multiple mouse models. CTLA-4 KO leads to lymphoproliferative disorders and early lethality, and PD-1 gene abrogation promotes severe autoimmune diseases, while TIM-3 KO and TIGIT KO cause experimental autoimmune encephalomyelitis (EAE) [15]. Pharmacological blockade of IC receptors exacerbates autoimmune events in mouse models of autoimmune diseases [15]. Hence, IC blockade therapies in cancer patients can lead to the appearance of irAEs, which can be variable from person to person. Typically, these toxicities, which have recently been reviewed [144], affect barrier tissues such as the skin, gastrointestinal tract, liver, and respiratory epithelium.

The overall response rate (ORR) to IC blockade antibodies, as a measure of therapy response, varies between tumor types (Table 3). This variation may be due to tumor-specific biological differences. Tumor types with a higher mutation rate and that conserve the expression of HLA have more neo-antigens, and are more likely to be identified and attacked by T cells. Moreover, tumor types with an immunosuppressive TME, including the presence of M2 macrophages, MDSCs, Treg cells, and immunosuppressive soluble factors, are less responsive to anti-IC therapies [145]. In addition, differences in ORR within same tumor type between drugs targeting the same pathway also arise (Table 3), possibly as a consequence of the intertumoral heterogeneity observed in patients with the same tumor type [146]. As previously described, IC blockade therapy response is affected by the TME profile, which also varies between patients [1].

To improve efficacy, the immune infiltrate and IC ligand expression in tumor and tumor-infiltrating immune cells could be characterized to determine the IC pathway most likely to be responsible for the attenuation of cytotoxic immune cell activity. Despite this, the overall response rates to single IC blockade therapies are generally low. Combining the blockade of multiple ICs is a strategy to increase the response of these therapies against certain tumor types but potentially increases the risk of irAE development. In order to minimize the risk of irAEs and maximize the response, the combinatory blockade of ICs with non-redundant downstream signaling could be a good strategy for enhancing antitumor immunity. Ideally, the blockade of an IC that recruits the phosphatases SHP-1 and/or SHP-2, such as PD-1, KLRG1, PVRIG, 2B4, Siglec-7/− 9, ILT2, TIGIT, NKG2A, KIR-L, or BTLA, should be combined with the blockade of another IC that exhibits alternative downstream signaling, such as CTLA-4, TIM3, LAG-3, CD47, CD200R1, or VISTA. Non-redundant IC combinatory blockade therapies may have synergistic effects that boost antitumoral immunity. However, further studies should be carried out to address this hypothesis. In addition, it needs to be considered that combining the blockade of any IC with CTLA-4 might present stronger secondary events given the role of CTLA-4 in regulating central tolerance [147]. Moreover, combinatory treatments that promote T and NK cell function simultaneously to reduce the presence of immunosuppressive cells might also be of value. The use of novel small-molecule inhibitors of PD-1/PD-L1 currently under clinical development might be beneficial because of their reduced immunogenicity. Indeed, immune checkpoint antibodies have a longer pharmacokinetic half-life than small-molecule inhibitors, which manifests as sustained immune system activation and a greater quantity of derived irAEs [148]. Combining small-molecule inhibitors of ICs concomitantly with the blockade of antibodies for different ICs may result in increased effector cell function and reduced tumor growth without any more frequent occurrence of irAEs.

Finding the correct IC blockade combination for each tumor setting to ensure that efficacy is increased without raising the risk of irAEs occurring is a major challenge. Understanding the mechanisms leading to irAEs will allow biomarkers to be developed that can classify patients according to the administration of the most effective and safe IC blockade therapy. Some of the plausible biomarkers studied for this purpose include peripheral blood cell counts, circulating cytokines and chemokines, the presence of autoantibodies, and the composition of gut microbiota [149]. However, the predictive information of these biomarkers has been studied in very few tumor types. The study of genetic polymorphisms associated with autoimmune diseases may also be of importance in identifying patients who are more likely to develop irAEs. Omics studies are of particular interest when reliable biomarkers across multiple tumor types need to be established. The expression of the lymphocyte cytosolic protein 1 (LCP1) and the adenosine diphosphate- dependent glucokinase (ADPGK) have been proposed as biomarkers of irAEs [150]. Further prospective trials are needed to identify the probable combinations of biomarkers that will allow us to categorize patients with respect to the determining the therapy that is safest for them to receive.

## Conclusions

The stimulation of antitumoral immunity through immunotherapy has revolutionized cancer treatment in recent years. Antibodies against CTLA-4, PD-1, and PD-L1 have been approved for the treatment of several types of tumors but have been of limited clinical benefit in some patients. This could be related to the existence of the many mechanisms that tumor cells use to evade the immune response, such as the expression of a long repertoire of IC ligands and the infiltration of several immunosuppressive immune cell populations. Blockade of novel ICs is being evaluated in clinical trials. Antibodies against LAG-3, TIM-3, TIGIT, CD47, and B7-H3 are the most advanced IC blockade drugs and may be approved for the treatment of specific tumor types in the near future, depending on the results of the trials. However, other challenges need to be overcome to fully exploit the therapeutic potential of blockade ICs, and thereby boost antitumoral immunity.

A full understanding of the crosstalk between cancer cells and the TME of every tumor type is needed to identify the specific immune-evasion mechanisms exploited by cancer cells and, subsequently, to apply proper therapy. Tumor cells can upregulate the expression of various IC ligands and promote the activation of multiple IC receptors of tumor-infiltrating immune cells. Hence, activation of alternative IC signals in the tumors may diminish the effect of single-blockade antibodies. Combinatory IC blockade treatments might present synergistic antitumoral responses in specific tumor types, and potentially increase the risk of secondary events such as irAEs. Hence, finding the best IC blockade combination to achieve increased effectiveness while minimizing the risk of irAEs should be a priority. Given that some IC receptors share downstream mechanisms to interfere with T and NK cell activation, the co-blockade of IC with non-redundant signaling to improve antitumoral immunity and prevent overlapping signals could be a good strategy. Furthermore, the use of small-molecule IC inhibitors might be advantageous compared with blockade antibodies, given their reduced immunogenicity. Importantly, development and analysis of biomarkers that allow patients to be classified according to their specific pathology settings and IC activation status should improve response rates to IC blockade therapies. The rational blockade of ICs, based on the specific tumor characteristics of each patient, may represent a breakthrough in our pursuit of a more personalized medicine.

## Supplementary Information

**Additional file 1: Supplementary Table 1.** Phase III clinical trials of single-blockade immune checkpoint inhibitors including CTLA-4, PD-1, and PD-L1 in 2021, according to www.clinicaltrials.gov.

## Data Availability

The datasets supporting the conclusions of this article are available in the Drug Approvals and Databases FDA repository, www.fda.gov and in the Data clinical trials repository, www.clinicaltrials.gov.

## Abbreviations

ICs: Immune checkpoints
CTLs: Cytotoxic T lymphocytes
NK: Natural killer
CTLA-4: Cytotoxic T lymphocyte-associated molecule-4
PD-1: Programmed cell death receptor-1
PD-L1: Programmed death ligand-1
PD-L2: Programmed cell death 1 ligand 2
MHC: Major histocompatibility complex
MDSCs: Myeloid-derived suppressor cells
Treg: T regulatory
TME: Tumor microenvironment
CAR: Chimeric antigen receptor
IFN: Interferon
TNF: Tumor necrosis factor
TCR: T-cell receptor
HLA: Human leukocyte antigen
APCs: Antigen-presenting cells
DCs: Dendritic cells
Th: T-helper
Th1: T-helper 1
Th2: T-helper 2
GM-CSF: Granulocyte-macrophage colony-stimulating factor
TILs: Tumor-infiltrating lymphocytes
FDA: US Food and Drug Administration
EMA: European Medicines Agency
KO: Knockout
mAb: Monoclonal antibody
CRC: Colorectal cancer
RCC: Renal cell cancer
NSCLC: Non-small cell lung cancer
LAG-3: Lymphocyte activation gene-3
FGL-1: Fibrinogen-like protein 1
TIM-3: T cell immunoglobulin and mucin domain-containing protein 3
PS: Phosphatidylserine
CEACAM1: Carcinoembryonic antigen-related cell adhesion molecule 1
HMGB1: High-mobility group protein 1
TIM-3: T-cell immunoglobulin and mucin-domain containing-3
TIGIT: T cell immunoglobulin and ITIM domain
PVRIG: Poliovirus receptor-related immunoglobulin
PVR: Poliovirus receptor
TACTILE: T cell activation increased late expression protein
KIR: Killer immunoglobulin-like
NKG2A: Natural killer group 2A
ILT-2: Immunoglobulin-like transcript-2
KIR-L: Long-tailed KIR
KIR-S: Short-tailed KIR
AML: Acute myeloid leukemia
IAP: Integrin-associated protein
SIRPα: Signal regulatory protein alpha
TSP1: Thrombospodin-1
HNSCC: Head and neck squamous cell carcinoma
Siglecs: Sialic acid-binding immunoglobulin type lectins
LPS: Lipopolysaccharide
KLRG1: Killer cell lectin-like receptor subfamily G member 1
BTLA: B and T cell lymphocyte attenuator
HVEM: Herpesvirus entry mediator
VISTA: V-domain Ig suppressor of T cell activation
PD-1H: Programmed death.1 homolog
PSGL-1: P-selectin glycoprotein ligand-1
B7-H3: B7 homolog 3 protein
EAE: Experimental autoimmune encephalomyelitis
ORR: Overall response rate
mPFS: Median progression-free survival
