# Supplementary material for: Blockade of novel immune checkpoints and new therapeutic combinations to boost antitumor immunity
Source: J Exp Clin Cancer Res. 2022 Feb 14;41:62. doi: 10.1186/s13046-022-02264-x (PMC8842574; doi:10.1186/s13046-022-02264-x)
Supplement: Supplementary file 1 — Additional file 1: Supplementary Table 1. Phase III clinical trials of single-blockade immune checkpoint inhibitors including CTLA-4, PD-1, and PD-L1 in 2021, according to www.clinicaltrials.gov. [file 13046_2022_2264_MOESM1_ESM.docx]

**Supplementary table 1. Phase III clinical trials of single-blockade immune checkpoint inhibitors including CTLA-4, PD-1, and PD-L1 in 2021, according to** [**www.clinicaltrials.gov**](http://www.clinicaltrials.gov)**.**

| **Target** | **Drug name** | **Indication** | **Status** |
| --- | --- | --- | --- |
| **PD-1** | Nivolumab | Bladder cancer; MIBC (NCT04209114); epithelial ovarian cancer; fallopian tube cancer (NCT03522246); gastric cancer; gastroesophageal cancer (NCT02872116); esophageal cancer (NCT02569242); recurrent glioblastoma multiforme (NCT02017717); glioblastoma multiforme (NCT02667587); refractory multiple myeloma (NCT02726581); malignant pleural mesothelioma (NCT02899299); HNSCC (NCT03576417); prostate cancer (NCT04100018); metastatic hormone-sensitive prostate cancer (NCT03879122) | Phase III |
| **PD-1** | Pembrolizumab | Metastatic hormone-sensitive prostate cancer (NCT04191096); prostate cancer (NCT03834493); muscle-invasive bladder cancer; urothelial cancer (NCT03244384); bladder cancer (NCT03711032); bile duct cancer (NCT04003636); CRC (NCT02563002); esophageal cancer (NCT03881111); ovarian cancer; fallopian tube cancer; peritoneal cancer (NCT03740165); biliary tract cancer (NCT04003636); malignant pleural mesothelioma (NCT02991482); RCC (NCT03142334); metastatic biliary tract cancer (NCT04003636); metastatic nasopharyngeal cancer (NCT02611960) | Phase III |
| **PD-1** | Cemiplimab | NSCLC (NCT03409614); metastatic NSCLC (NCT03088540); cervical cancer (NCT03257267) | Phase III |
| **PD-1** | Camrelizumab | Hodgkin lymphoma (NCT04342936); gastric cancer (NCT04342910); metastatic nasopharyngeal cancer (NCT03707509); NSCLC (NCT04203485); nasopharyngeal cancer (NCT03427827); GEJ cancer (NCT04208347); esophageal cancer (NCT03691090) | Phase III |
| **PD-1** | Sintilimab | ESCC (NCT03748134); nasopharyngeal carcinoma (NCT03700476); NSCLC (NCT03884192); gastric cancer (NCT03745170); metastatic squamous NSCLC (NCT03629925); metastatic CRC (NCT04194359); Hodgkin lymphoma (NCT04044222); SCLC (NCT04192682); rectal cancer (NCT04304209); HCC (NCT04167293); metastatic NSCLC (NCT03150875) | Phase III |
| **PD-1** | Tislelizumab | Squamous NSCLC (NCT03594747); metastatic nasopharyngeal cancer (NCT03924986); metastatic gastric cancer; metastatic GEJ cancer (NCT03777657); NSCLC (NCT03745222); ESCC (NCT03430843); urothelial cancer (NCT03967977); SCLC (NCT04005716); metastatic); ESCC (NCT03783442) | Phase III |
| **PD-1** | Toripalimab | HCC (NCT03949231); HNSCC (NCT03952065); metastatic nasopharyngeal cancer (NCT04376866); ESCC (NCT03829969); SCLC (NCT04012606); NSCLC (NCT04158440); TNBC (NCT04085276); extranodal natural killer/T-cell lymphoma (NCT04365036); metastatic melanoma (NCT03430297); metastatic neuroendocrine carcinoma of the bladder (NCT03992911); esophageal carcinoma (NCT04280822) | Phase III |
| **PD-1** | Dostarlimab | Endometrial cancer (NCT03981796); epithelial ovarian cancer (NCT03602859); ovarian carcinosarcoma; endometrial carcinosarcoma (NCT03651206) | Phase III |
| **PD-1** | CS-1003 | HCC (NCT04194775) | Phase III |
| **PD-1** | HLX-10 | Gastric cancer (NCT04139135); TNBC (NCT04301739); metastatic ESCC (NCT03958890); squamous NSCLC (NCT04033354); SCLC (NCT04063163); metastatic NSCLC (NCT03952403) | Phase III |
| **PD-1** | PF-06801591 | NMIBC (NCT04165317) | Phase III |
| **PD-1** | Spartalizumab | Metastatic melanoma (NCT02967692) | Phase III |
| **PD-1** | REGN2810 | Metastatic NSCLC (NCT03088540); metastatic cervical cancer (NCT03257267); NSCLC (NCT03409614); cSCC (NCT03969004) | Phase III |
| **PD-1** | AK105 | HCC (NCT04344158); metastatic squamous NSCLC (NCT03866993) | Phase III |
| **PD-1** | INCMGA00012 | Metastatic squamous NSCLC; metastatic NSCLC (NCT04205812); NSCLC (NCT04203511); HNSCC (NCT04129320); gastric cancer; GEJ cancer (NCT04082364) | Phase III |
| **PD-L1** | Atezolizumab | Squamous NSCLC (NCT02367794); NSCLC (NCT02409342); HCC (NCT03755791); metastatic HCC (NCT03434379); TNBC (NCT03281954); breast cancer (NCT03726879); melanoma (NCT02908672); metastatic cervical cancer (NCT03556839); RCC (NCT03024996); metastatic RCC (NCT03693573); metastatic bladder cancer (NCT02302807); ovarian cancer (NCT03353831); fallopian tube cancer; peritoneal cancer (NCT03038100); HNSCC (NCT03452137); endometrial cancer (NCT03603184); malignant pleural mesothelioma (NCT03762018) | Phase III |
| **PD-L1** | Durvalumab | Hypopharyngeal cancer; laryngeal cancer; oral cavity cancer; oropharyngeal cancer (NCT03258554); RCC (NCT03288532); cervical cancer (NCT03830866); metastatic HNSCC (NCT02551159); HNSCC (NCT03258554); bladder cancer (NCT03528694); MIBC (NCT03732677); ovarian cancer (NCT03737643); HCC (NCT03298451); metastatic NSCLC (NCT02352948); malignant pleural mesothelioma (NCT04334759) | Phase III |
| **PD-L1** | Avelumab | Colon cancer (NCT03827044); TNBC (NCT02926196); gastric cancer (NCT02625623); HNSCC (NCT02952586); NSCLC (NCT02395172); GEJ (NCT02625610); ovarian cancer (NCT02580058); epithelial ovarian cancer (NCT02718417); diffuse large B-cell lymphoma (NCT02951156) | Phase III |
| **PD-L1** | CS1001 | Gastric cancer; GEJ cancer (NCT03802591); metastatic ESCC (NCT04187352); NSCLC (NCT03789604) | Phase III |
| **PD-L1** | Lazertinib | Metastatic NSCLC (NCT04248829); | Phase III |
| **PD-L1** | SHR-1316 | SCLC (NCT03711305); NSCLC (NCT04316364) | Phase III |
| **PD-L1** | Bintrafusp alfa | Metastatic biliary tract cancer (NCT04066491); NSCLC (NCT03631706) | Phase III |

NSCLC, non-small-cell lung cancer; GEJ, gastroesophageal junction; HNSCC, head and neck squamous cell carcinoma; SCLC, small-cell lung cancer; RCC, renal cell carcinoma; HCC, hepatocellular carcinoma; MIBC, muscle invasive bladder cancer; ESCC, esophageal squamous cell carcinoma; TNBC, triple-negative breast cancer; CRC, colorectal cancer; NMIBC, non-muscle invasive bladder cancer; cSCC, cutaneous squamous cell carcinoma; AML, acute myeloid leukemia.
